# A blood based 12-miRNA signature of Alzheimer disease patients

**DOI:** 10.1186/gb-2013-14-7-r78

**Published:** 2013-07-29

**Authors:** Petra Leidinger, Christina Backes, Stephanie Deutscher, Katja Schmitt, Sabine C Mueller, Karen Frese, Jan Haas, Klemens Ruprecht, Friedemann Paul, Cord Stähler, Christoph JG Lang, Benjamin Meder, Tamas Bartfai, Eckart Meese, Andreas Keller

**Affiliations:** 1 Department of Human Genetics, Saarland University, Kirrbergerstraße, Building 60, 66421 Homburg, Germany; 2 Internal Medicine II, Heidelberg University, Im Neuenheimer Feld 350, 69120 Heidelberg, Germany; 3 Clinical and Experimental Multiple Sclerosis Research Center, Charité - University Medicine Berlin, Campus Mitte, Charitéplatz 1, 10117 Berlin, Germany; 4 NeuroCure Clinical Research Center, Charité - University Medicine Berlin, Campus Mitte, Charitéplatz 1,10117 Berlin, Germany; 5 Siemens Healthcare, Strategy, Hartmannstr. 16, 91052 Erlangen, Germany; 6 Neurological Unit, University of Erlangen, Schwabachanlage 6, 91054 Erlangen, Germany; 7 Department of Chemical Physiology, The Scripps Research Institute, 10550 N Torrey Pines Rd, La Jolla, CA 92037, USA

**Keywords:** Alzheimer disease, miRNA, biomarker, next-generation sequencing, quantitative Real Time PCR

## Abstract

**Background:**

Alzheimer disease (AD) is the most common form of dementia but the identification of reliable, early and non-invasive biomarkers remains a major challenge. We present a novel miRNA-based signature for detecting AD from blood samples.

**Results:**

We apply next-generation sequencing to miRNAs from blood samples of 48 AD patients and 22 unaffected controls, yielding a total of 140 unique mature miRNAs with significantly changed expression levels. Of these, 82 have higher and 58 have lower abundance in AD patient samples. We selected a panel of 12 miRNAs for an RT-qPCR analysis on a larger cohort of 202 samples, comprising not only AD patients and healthy controls but also patients with other CNS illnesses. These included mild cognitive impairment, which is assumed to represent a transitional period before the development of AD, as well as multiple sclerosis, Parkinson disease, major depression, bipolar disorder and schizophrenia. miRNA target enrichment analysis of the selected 12 miRNAs indicates an involvement of miRNAs in nervous system development, neuron projection, neuron projection development and neuron projection morphogenesis. Using this 12-miRNA signature, we differentiate between AD and controls with an accuracy of 93%, a specificity of 95% and a sensitivity of 92%. The differentiation of AD from other neurological diseases is possible with accuracies between 74% and 78%. The differentiation of the other CNS disorders from controls yields even higher accuracies.

**Conclusions:**

The data indicate that deregulated miRNAs in blood might be used as biomarkers in the diagnosis of AD or other neurological diseases.

## Background

Alzheimer disease(AD) is the most common form of neurodegenerative illness leading to dementia which is predicted to affect as much as 1 in 85 people globally by 2050 [1]. While early-onset (familiar) AD has been reported in younger people, the majority of (sporadic) AD cases is diagnosed in people aged over 65 years [2]. As of today, final diagnosis of AD can only be achieved by autopsy making the identification of reliable, early, and non-invasive biomarkers a major challenge. Finding such non-invasive, reliable diagnostic tools is of paramount importance as it appears that early intervention in the prodromal stage of AD or the identification and therapy of those patients with mild cognitive impairment who will transform to AD rapidly might be a possibility to delay the onset of AD substantially [3].

A prominent example of recently developed AD biomarker assays is the combinatorial analysis of the concentration of peptides and proteins: beta-amyloid-1-42 (Aß 42), tau, and/or p-tau in the cerebrospinal fluid (CSF). According to the S3 guidelines, an increased level of tau protein together with a decreased level of beta-amyloid-1-42 provides strong evidence for the presence of AD [4]. The combinatorial analysis of all three factors yields even higher diagnostic accuracy than the combination of only two of the above-mentioned proteins [5]. Furthermore, combinatorial analysis of Aß levels and tau levels can discriminate between patients with stable mild cognitive impairment (MCI) and patients with progressive MCI into AD or other types of dementia with a sufficient diagnostic accuracy [6]. Nevertheless, according to the S3 guidelines, the analysis of CSF biomarker is only indicated to confirm the diagnosis if other clinical symptoms give evidence for the presence of neurodegenerative dementia or for the differential diagnostics of other forms of diseases that can cause symptoms like dementia (encephalitis, neuroborreliosis, multiple sclerosis, Lues, brain abscess, metastases).

The use of peripheral markers, like Aß and tau in easily accessible peripheral cells (in particular platelets and skin fibroblasts), as a diagnostic tool has been under investigation for more than 10 years [7,8]. Molecular genetics analyses of common single nucleotide polymorphisms (SNPs) in genes such as presenilin or ApoE4 did not significantly improve risk estimation for the susceptibility of AD [9]. Likewise, there is no consistent evidence for an association between AD and genetic variation of mitochondrial DNA (mtDNA) [10].

There is increasing effort to develop molecular diagnostic markers that meet requirements like easy accessibility, for example, from blood, sufficiently high specificity and sensitivity, low costs and applicability by laboratories with standard equipment. Several blood, plasma, or serum born AD biomarkers have been proposed to meet these criteria. Doecke et al. recently presented a panel of protein biomarkers to reliably detect AD with an accuracy of 85% [11]. Moreover, Tan et al. provided evidence that the proteins p53 and p21 can be used to detect AD using blood samples. A receiver operating characteristic curve analysis revealed a specificity of 76% and a sensitivity of 84% for p53, 88% and 82% for p53(ser15), 80% and 75% for p21, and 84% and 68% for p21(thr145) [12].

Besides proteins microRNAs (miRNAs) have also demonstrated their potential as non-invasive biomarkers from blood and serum for a wide variety of human pathologies [13]. A deregulation of miRNA expression might be involved in neurological dysfunction or neurodegenerative processes. Interestingly, Liang et al. [14] showed that the expression pattern of brain and blood PBMC cluster together which might be an indication that a specific blood based expression signature might prove to be useful as biomarker for AD and other neurological diseases. MiRNA expression analyses can be readily applied for *in vitro *diagnostic testing by molecular diagnostics and CLIA (Clinical Laboratory Improvement Amendments) laboratories.

While altered miRNA patterns have been exhaustively investigated in AD patients' tissue samples or cell cultures [15-18], less information on circulating miRNAs in AD is known. A recent serum profiling of AD patients provided first evidence that expression changes of circulating miRNAs may be valuable biomarkers for AD [19].

We describe our results obtained by applying the next-generation sequencing (NGS) approach to screen the expression of all human miRNAs in blood from extensively characterized AD patients and healthy controls. Patient blood was obtained from the SAMPLE (Serial Alzheimer diseaseand MCI Prospective Longitudinal Evaluation) Registry of PrecisionMed (San Diego, CA, USA) and blood from age-matched healthy donors from the ACE (Aging Cognition Evaluation) Registry, a PrecisionMed- UBC (The University of British Columbia) collaboration. We identified 140 unique differentially expressed miRNAs between AD patients and controls. Validation of a 12-miRNA signature was carried out by RT-qPCR in a cohort of 202 samples encompassing patients suffering from other neurological disorders including mild cognitive impairment as a potential preliminary stage of AD, and other neurodegenerative diseases like Parkinson disease and multiple sclerosis as well as mental diseases like schizophrenia (SCHIZ), major depression (DEP), and bipolar disorder (BD).

A combination of AD-specific miRNA expression signatures with the rapidly developing and expanding amyloid load imaging techniques may be useful as non-invasive diagnostic tools in AD diagnosis in the future [20].

## Results

### Initial biomarker screening using next-generation sequencing

To detect potential AD biomarkers we examined blood from well-characterized patients and controls. We obtained blood from the SAMPLE (Serial Alzheimer diseaseand MCI Prospective Longitudinal Evaluation) Registry of PrecisionMed (San Diego, CA, USA). SAMPLE is a sample depository resulting from a longitudinal study that evaluates cognition in women and men, who are recruited, evaluated, cognitively studied, and sampled from 12 to 15 experienced investigative sites in USA. All participants underwent several tests (that is, Alzheimer Disease Assessment Scale-cognitive subscale (ADAS-Cog), Clinical Dementia Rating (CDR), Wechsler Memory Scale, and Mini-Mental State Exam (MMSE)) to evaluate cognition. Blood from age-matched healthy donors was obtained from the Ace Registry, which is a biological sample bank of serial patient samples with linked serial cognition data, based on a cognition battery selected from UBC's proprietary computerized testing platform.

We carried out high-throughput NGS of 22 healthy control samples (C) and 48 AD patient samples using IlluminaHiSeq 2000 sequencing with eight multiplexed samples on each sequencing lane. We detected not only known human miRNAs, but also novel miRNA candidates that have previously not been included in the miRBase v18 [21,22]. These miRNA candidates are, however, much less abundant compared to the known human miRNAs. After removing the least abundant miRNAs (that is, all miRNAs with <50 read counts summed up across all samples of each group) we detected a total of 383 different miRNA precursors resulting in 416 unique mature miRNA forms.

To compare the NGS results of the AD patient samples with the samples from healthy donors we first computed Wilcoxon-Mann-Whitney (WMW) test and adjusted the significance values for multiple testing using Benjamini-Hochberg adjustment. All miRNAs with adjusted significance values <0.05 were considered statistically significant. We also computed the area under the receiver operator characteristics curve (AUC). In total, we detected 180 significantly dys-regulated miRNAs (140 unique mature miRNAs) including 90 miRNAs (58 unique mature miRNAs) that were downregulated and 90 miRNAs (82 unique mature miRNAs) that were upregulated in AD samples compared to healthy control samples (see Additional file 1-Table S1). Additional file 2-Figure S1 shows a heatmap for 180 significantly dys-regulated miRNAs. The most upregulated miRNA was hsa-miR-30d-5p (AUC of 0.0819) with a *P*value of 8.35*10^-6^and the most downregulated miRNA was hsa-miR-144-5p (AUC of 0.9138) with *P*value of 8.35*10^-6^. While the high AUC value indicates that each of these miRNAs has sufficient power to differentiate between AD and healthy controls, they are not specific for AD since both miRNAs have already been described for many other human pathologies, including different neoplasms [13]. Among the significantly dys-regulated miRNAs are also 15 novel miRNA candidates (called brain-miR) that were all upregulated in AD compared to controls. A list of all novel mature miRNAs is provided in Additional file 3-Table S2. To gain first insight into the biological function of the mature miRNAs that were dys-regulated between AD patients and healthy control individuals, we applied a miRNA over-representation analysis for these miRNAs using the TAM (tool for annotations of human miRNAs) database [23,24]. The TAM database classifies over- or under-represented miRNAs according to the categories miRNA family, miRNA cluster, miRNA function, miRNA associated diseases, and tissue specificity. We detected for all dys-regulated miRNAs 56 significant categories (*P*value <0.05 after adjustment for multiple testing), with the interesting categories miR-30 family with five miRNAs being upregulated (*P *value 6.64*10^-4^), the let-7 family with nine downregulated miRNAs (*P*value 5.65*10^-7^), and the disease category Alzheimer disease for which six dys-regulated miRNAs were relevant, including hsa-miR-21, hsa-miR-17, hsa-miR-29a, hsa-miR-29b, hsa-miR-106b, and hsa-miR-107 (*P*value 0.0139).

To determine whether the 140 unique differentially expressed miRNAs between AD patients and healthy controls cluster together within a same genomic region, which would suggest presence of common regulatory mechanisms for their expression, we sorted all miRNAs according to their position on each chromosome. Then, we assigned the miRNAs to one of the following three classes: not dys-regulated; upregulated in AD; and downregulated in AD. Finally, we searched for regions that contain at least three different dys-regulated mature miRNAs by applying window sizes varying between 1,000 and 100,000 base pairs. Within regions encompassing <1,000 base pairs we detected two clusters including one on chromosome 19 with the upregulated miRNAs hsa-miR-99b-5p and hsa-miR-125a-5p and the downregulated miRNA hsa-let-7e-5p and a second cluster on chromosome 22 with the downregulated miRNAs hsa-let-7a-5p and hsa-let-7b-5p and the upregulated miRNA hsa-let-7b-3p. Analyzing regions of up to 5,000 base pairs, we found on chromosome 9 a dense cluster with a total of five dys-regulated miRNAs including the downregulated miRNAs hsa-let-7a-5p, hsa-let-7f-5p, and hsa-let-7d-5p and the upregulated miRNAs hsa-let-7f-1-3p and hsa-let-7d-3p. For regions up to 30,000 base pairs, we discovered one region on chromosome 6 with three co-located miRNAs including hsa-miR-30c-5p, hsa-miR-30a-3p, and hsa-miR-30a-5p, all of which were upregulated. To understand whether the miRNAs are regulated by specific transcription factors (TF), we extracted potential TF binding sites from the UCSC genome browser but found no evidence for a significant enrichment for specific TF binding sites.

**Figure 1:**
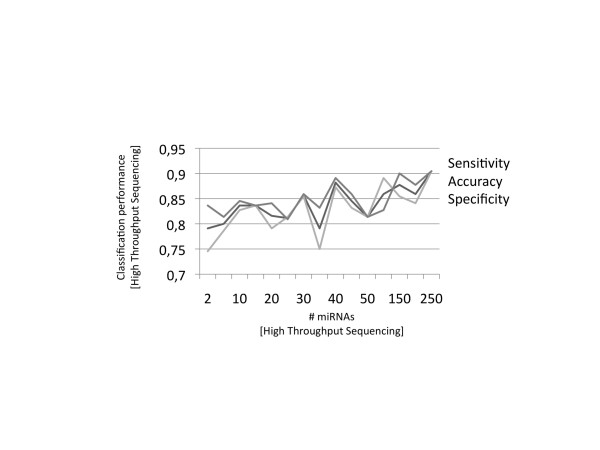
**Classification performance dependent on miRNA combinations**. With increasing number of miRNAs the accuracy, specificity, and sensitivity increases towards convergence at 90%.

In the next step, we performed classification of AD and control samples using a standard machine learning approach. In a cross-validation loop, we stepwise added the miRNAs with lowest significance values and repeatedly carried out radial basis function support vector machines (SVM). As shown in Figure 1, a signature of 250 miRNAs yields an accuracy, specificity, and sensitivity of 90%. Since this set of miRNAs contains a significant amount of redundant miRNAs with largely identical information and high correlation among many miRNAs, a significantly smaller set of miRNAs is likely to yield comparably accurate distinction between AD samples and samples from healthy controls. We selected 12 miRNAs with limited cross-correlation, including strongly dys-regulated miRNAs that show a potential to separate AD from controls. We furthermore compared our NGS results with previous studies on different types of cancer and non-cancer diseases [13] in order to ensure that the selected miRNAs are not dys-regulated in several other diseases. Besides known miRNAs we also included two unknown miRNAs, namely brain-miR-112 and brain-miR-161. Finally, the selected 12-miRNA signature contains the miRNAs brain-miR-112, brain-miR-161, hsa-let-7d-3p, hsa-miR-5010-3p, hsa-miR-26a-5p, hsa-miR-1285-5p, and hsa-miR-151a-3p, all of which are upregulated in AD and the downregulated miRNAs hsa-miR-103a-3p, hsa-miR-107, hsa-miR-532-5p, hsa-miR-26b-5p, and hsa-let-7f-5p.

### Validation of a 12-miRNA signature by RT-qPCR

To validate the 12-miRNA signature we employed RT-qPCR and included not only additional patients with AD, but also patients with other diseases including neurological disorders. In total, we analyzed 12 miRNAs in 202 samples as detailed in Table 1.

**Table 1:** Overview of the blood samples analyzed using NGS and RT-qPCR

Sample group	*N*	Age (mean ± SD)	Sex (female/male)	MMSE (mean ± SD)	Cohort size NGS	Cohort size RT-qPCR
AD	106	72.7(10.4)	53/53	18.9 (3.4)	48	94

Healthy control	22	67.1 (7.5)	11/11	29.3 (1.2)	22	21

Mild cognitive impairment	18	73.9 (6.2)	9/9	25.3 (1.4)	-	18

Multiple sclerosis	16	32.3 (10.7)	12/4	NA	-	16

PD	9	69.7 (9.0)	1/8	25.2 (4.2)	-	9

DEP	15	45.2 (9.1)	0/15	NA	-	15

BD	15	41.9 (13.7)	14/1	29.5 (1.6)	-	15

SCHIZ	14	41.7 (7.9)	1/13	26.1 (4.3)	-	14

AD: Alzheimer disease; BD: bipolar disorder; DEP: major depression; MMSE: Mini-Mental State Exam; NA: not available; NGS: next-generation sequencing; PD: Parkinson's disease; RT-qPCR: quantitative real-time PCR; SCHIZ: schizophrenia.

**Figure 2:**
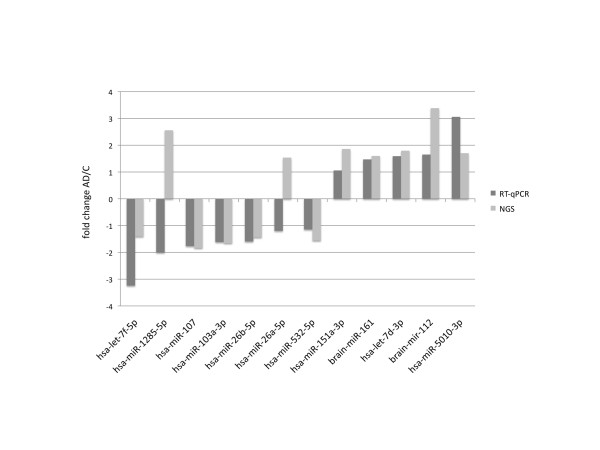
**Comparison of the expression analysis results of AD patients *versus *healthy controls (fold changes) obtained by NGS and RT-qPCR for the 12-miRNA signature**.

We first considered the miRNA fold quotients that were obtained for AD samples and controls. We compared the fold quotients of each of the 12 miRNAs between initial NGS screening cohort and the RT-qPCR validation cohort. All but two of the 12 miRNAs, namely hsa-miR-1285-5p and hsa-miR-26a-5p, have been dys-regulated in the same direction in both approaches, indicating a high degree of concordance between screening and validation study. Both hsa-miR-1285-5p and hsa-miR-26a-5p have been significantly upregulated in AD in the NGS screening experiment while downregulated in the RT-qPCR validation (see Figure 2). This discrepancy might be due to the duplication of the AD sample cohort. However, SVM classification on the RT-qPCR data to separate AD and controls using linear kernels in 10-fold cross-validations with 100 repetitions reached on average an accuracy of 93.3%, a specificity of 95.1%, and a sensitivity of 91.5%. The computed means, standard deviations, and confidence intervals for the repetitions concerning specificity, sensitivity, and accuracy are presented in Table 2, as well as the results for the control classifications with the randomly permuted class labels.

**Table 2:** Summary of the SVM classifications containing the means, standard deviations, and 95% confidence intervals (CI) of the accuracy (acc), specificity (spec), sensitivity (sens) running 100 repetitions of 10-fold cross-validations with linear kernel.

Comparison	Classification			Permutation test		
	Acc	Spec	Sens	Acc	Spec	Sens

AD *vs*. control	93.3% ± 4.6 CI:92.4-94.2%	95.1% ± 5.4 CI:94.1-96.2%	91.5% ± 5.8 CI:90.4-92.7%	50.7% ± 12.5 CI:48.2-53.1%	50.7% ± 13.3 CI:48.1-53.3%	50.7% ± 14.1 CI:47.9-53.4%

MCI *vs*. control	84.2% ± 3.7 CI:83.4-84.9%	81.1% ± 5.6 CI:80.0-82.2%	87.7% ± 3.7 CI:87.0-88.5%	51.3% ± 11.4 CI:49.0-53.5%	52.0% ± 12.2 CI:50.0-54.4%	50.4% ± 13.5 CI:47.8-53.1%

PSY *vs*. control	97.1% ± 1.6 CI:96.8-97.4%	95.3% ± 1.7 CI:95.0-95.6%	99.0% ± 2.4 CI:98.5-99.4%	48.7% ± 10.6 CI:46.7-50.8%	48.5% ± 12.4 CI:46.0-50.9%	49.0% ± 12.1 CI:46.6-50.8%

Other ND *vs*. control	82.8% ± 5.0 CI:81.8-83.7%	84.0% ± 5.8 CI:83.0-85.2%	81.4% ± 6.7 CI:80.1-82.7%	50.3% ± 10.3 CI:48.3-52.3%	50.7% ± 11.7 CI:48.4-53.0%	50.0% ± 12.0 CI:47.6-52.3%

NEURO *vs*. control	86.1% ± 5.7 CI:85.0-87.2%	88.7% ± 6.8 CI:87.3-90.0%	83.6% ± 6.6 CI:82.3-84.9%	49.9% ± 10.8 CI:47.8-52.1%	50.1% ± 11.5 CI:47.9-52.3%	49.8% ± 13.3 CI:47.2-52.3%

AD *vs*. MCI	75.6% ± 7.8 CI:74.1-77.2%	76.7% ± 8.3 CI:75.1-78.4%	74.6% ± 9.7 CI:72.7-76.5%	50.6% ± 9.4 CI:48.7-52.4%	51.2% ± 10.4 CI:49.1-53.2%	49.9% ± 11.7 CI:47.7-52.2%

AD *vs*. PSY	77.8% ± 4.0 CI:77.0-78.5%	76.3% ± 4.8 CI:75.4-77.3%	79.2% ± 5.4 CI:78.1-80.2%	50.0% ± 8.0 CI:48.5-51.6%	49.1% ± 9.3 CI:47.3-50.9%	51.1% ± 10.3 CI:49.1-53.1

AD *vs*. other ND	73.8% ± 4.4 CI:72.9-74.7%	75.2% ± 4.7 CI:74.2-76.1%	72.4% ± 6.4 CI:71.2-73.7%	50.1% ± 7.3 CI:48.7-51.5%	49.2% ± 9.4 CI:47.4-51.1%	51.0% ± 8.5 CI:49.3-52.7%

The right part of the table contains the results for the permuted class labels. PSY = psychological disorders (DEP, BD, SCHIZ), other ND = other neurodegenerative disorders (PD, MS, MCI), NEURO = PSY + other ND

To evaluate whether the selected miRNAs are stage-dependent we further grouped the AD patients according to their MMSE score into mild AD (MMSE >19, *n *= 39) and moderate AD (MMSE 12-19, *n *= 46). The MMSE is a short test of 30 questions used to screen for cognitive impairment. Each question to be answered is scored with points, with a maximum possible score of 30 points. This questionnaire can be used to estimate the severity of cognitive impairment and to follow the course of cognitive changes in an individual over time. Normally, patients reaching 27 to 30 points do not suffer from dementia, 10 to 26 points are indicative for mild-to-moderate dementia, and less than 9 points indicates severe dementia. We found no significant expression differences of the 12-miRNA signature between the mild AD group and the moderate AD group.

**Figure 3:**
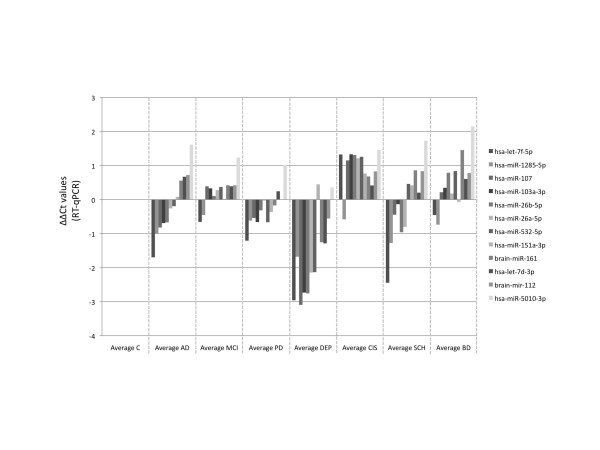
**Results of the RT-qPCR analysis of the 12 miRNAs in each of the analyzed diseases**. ΔΔCT values of the patients groups as compared to controls are shown on the y-axis. A lower expression in the patients than in controls is indicated by the bars <0 and higher expression in the patients as compared to controls is indicated by the bars >0. (C = healthy control, AD = Alzheimer disease, MCI = mild cognitive impairment, PD = Parkinson disease, DEP = major depression, CIS = multiple sclerosis (clinically isolated syndrome), SCH = schizophrenia, BD = bipolar disorder).

As patients with other neurological disorders can show similar symptoms as AD patients, we decided to validate our AD NGS results also with samples from patients with several neurological diseases. Specifically, we asked whether other neurological disorders show significant deviations in the expression of the 12 miRNAs. The results of this validation help to determine whether the investigated miRNAs have the potential for clinical applications. We analyzed patients with neurodegenerative diseases (MCI, Parkinson disease (PD), multiple sclerosis (clinically isolated syndrome, CIS)) and patients with psychiatric disorders (SCHIZ, BD, and DEP) for the signature of 12 miRNAs. The pattern, which was closest to AD was SCHIZ, where we found six up- and six downregulated miRNAs. We found a strong overall downregulation for most of the selected 12 miRNAs for patients with DEP and PD and a strong overall upregulation for patients with MCI, CIS, and BD (Figure 3).

In addition, we also applied machine learning procedures using SVM to estimate the accuracy, sensitivity, and specificity of the 12-miRNA signature regarding the other neurological diseases in comparison to the control group and to AD. The results of these classifications are also listed in Table 2. Interestingly, while the 12 miRNAs were chosen for their potential to separate AD and controls, this signature also separates the group of the psychological disorders (DEP, BD, SCHIZ) from controls with an accuracy of 97.1%, a specificity of 95.3%, and a sensitivity of 99.0% whereas other neurodegenerative diseases (PD, multiple sclerosis, mild cognitive impairment) were separated from controls with a worse accuracy of 82.8%, a specificity of 84.0%, and a sensitivity of 81.4%. The average accuracy for the other classifications against controls (that is, MCI *versus *control and neurodegenerative and psychological disorders *versus *control) reached values of 84.2% and 86.1%, respectively. Furthermore, we tested how well the 12-miRNA signature separates AD from MCI, AD from psychological disorders, and AD from other neurodegenerative diseases, respectively. The average accuracy for these comparisons was between 73.8% and 77.8%. Since the 12-miRNA signature has been tailored to differentiate between AD and controls, other miRNAs may likely contribute to a signature that permits also a better differentiation between the other tested diseases and AD.

### Prediction of miRNA targets and over-representation analysis

Target gene prediction of the 10 known miRNAs from the 12-miRNA signature revealed 2,354 genes that may be regulated by those miRNAs. These target genes were used to perform an over-representation analysis and identified 73 computed Gene Ontology (GO) categories with *P*values <0.05 and FDR adjustment. Interestingly, we found a significant enrichment of miRNA targets in the GO categories nervous system development, neuron projection, neuron projection development, and neuron projection morphogenesis. These GO categories are listed in Table 3 together with the predicted miRNA target genes involved in these categories. Furthermore, target genes that have already been related to AD or other neurological diseases are also listed in the table in separate columns.

**Table 3:** Results of the over-representation analysis of the predicted target genes of the 10 known miRNAs.

Subcategory	Subcategory alternative name	Expected	Observed	*P*value (FDR)	Target genes	AD	BD	DEP	PD	SCHIZ	Multiple sclerosis
Nervous system development	GO:0007399	159,555	215	0,000921692	ARSB ATM GJA1 JAG1 LEP NDP PTEN PAFAH1B1 TWIST1 DRD1 IGF1R GDF6 SMPD1 KCNMA1 NTRK2 CTNS NF1 INSC SLC6A3 FBXO45 IGF1 ADM APC DLG4 GRIN2A PAX7 PPT1 GPSM1 FEZF1 TSC1 DISC1 GLRB BMPR1B CDK6 CX3CR1 CELSR2 ID4 ERBB3 FGF2 AFF2 GLRA2 **GSK3B **HOXB3 LAMC1 LRP6 LSAMP NGF NPAS2 OPHN1 P2RY1 PEX13 POU3F1 PTPRZ1 SALL1 SMARCC1 STRN T TFAP2A TGFB2 TIAM1 NR2C2 YWHAH ZIC1 ULK1 ENC1 IRS2 ADAM23 KALRN SEMA5A EDNRB DMD AQP4 GMFB SDHA SLC1A2 GDA VCAN DVL1 EPHA4 EPHA7 KIF5C LRP2 POU4F2 RPS6KA3 SPOCK1 TGFBR1 AXIN2 DCLK1 MED1 ONECUT2 SIM1 CNTN2 ATF1 DLX6 ERBB4 SMAD4 SIX3 NHLH1 POU3F2 REST ABI2 PURA SMAD1 NAB1 SIX1 PPARD PRKCQ CHERP MAB21L2 TBR1 CHL1 FRS2 FKTN BTG2 SHOX2 SLC5A3 ZNF24 WWP1 STMN2 RAPGEF5 PIP5K1C ATXN10 RACGAP1 GREM1 NRG1 CNTNAP2 RPS6KA6 CYFIP1 ULK2 NLGN1 RUFY3 ARHGAP26 NFASC CLASP2 NIPBL SUFU PDGFC HPCAL4 RAPGEFL1 SHC3 FZD3 SIX4 BAIAP2 CSGALNACT1 PCDHB10 NMUR2 VANGL2 SEMA6A CNTN3 LRRC4C RET GNAO1 SCN2A FGF12 XRCC5 NTN4 BCR ADAM22 ACSL4 FGFR1 HTR5A NOTCH2 TTLL7 PGAP1 JHDM1D ATXN3 ZEB1 NDEL1 MAP2 B3GNT5 CHD6 SLITRK6 ELAVL3 HOOK3 ATOH8 WNT3A ZIC5 FGF1 SOX6 PDE5A SNAP25 GRIN3A CREB1 NRXN1 NRXN3 TPM3 FYN SEMA6D HOXA1 BDNF ALDH5A1 UNC5B DMBX1 IL6ST UHMK1 DCX CUX1 ATL1 GLDN RNF6 FAM5C CCNG1 NRP2 GAS7 ACSL3 RCAN1 SYNJ1 PCDH9 MOG RTN1 QKI LIG4 MBNL1 CCDC64 WNK1	DRD1 IGF1R GSK3B FGF1 FYN BDNF	DRD1 DISC1 GSK3B BCR HTR5A BDNF SYNJ1	BCR SNAP25 CREB1 BDNF	BDNF	LEP DRD1 SLC6A3 GRIN2A DISC1 YWHAH SLC1A2 CHL1 NRG1 FZD3 HTR5A SNAP25 BDNF SYNJ1	JAG1

Neuron projection	GO:0043005	49,9516	79	0,00411039	ADRB2 CA2 PAFAH1B1 ATP1A2 DRD1 GABRA6 GAD1 GRM3 IGF1R KCNJ2 NPY1R PGR AR KCNMA1 NF1 TACR1 MYO5B ACTN2 GRM1 APC GRIN2A ATXN1L MYO5A PPT1 OPRM1 TSC1 HTR2A CALCR OPHN1 STRN TGFB2 ULK1 PRSS12 KALRN BNIP3 SLC1A2 DVL1 EPHA7 KIF5C NCAM2 KIF5A CNTN2 ABI2 PURA CAPRIN1 IGF2BP1 SCN1A STMN2 SNCA STAT1 EPB41L3 ATXN10 CNTNAP2 RUFY3 NFASC ERC2 KIAA1598 SEMA6A SCN2A GAN TTLL7 CPEB1 NDEL1 MAP2 PSD2 CALD1 SNAP25 GRIN3A TPM3 AQP11 UHMK1 EXOC8 DICER1 ATL1 ANKS1B RNF6 CCNG1 CACNA1C NRP2	DRD1 IGF1R HTR2A SNCA	DRD1 HTR2A	HTR2A	SNCA	DRD1 GAD1 GRM3 GRIN2A HTR2A SLC1A2 SNAP25	ADRB2

Neuron projection development	GO:0031175	43,1466	67	0,0162232	GJA1 PTEN PAFAH1B1 IGF1R ADM APC FEZF1 DISC1 BMPR1B CELSR2 ERBB3 LAMC1 NGF OPHN1 PTPRZ1 STRN TIAM1 YWHAH ULK1 KALRN DMD VCAN DVL1 EPHA4 EPHA7 KIF5C POU4F2 DCLK1 CNTN2 ATF1 POU3F2 ABI2 SMAD1 TBR1 STMN2 PIP5K1C ATXN10 CYFIP1 ULK2 RUFY3 NFASC FZD3 BAIAP2 SEMA6A LRRC4C GNAO1 ACSL4 FGFR1 NDEL1 MAP2 SLITRK6 WNT3A SNAP25 GRIN3A CREB1 NRXN1 NRXN3 HOXA1 BDNF UNC5B UHMK1 DCX ATL1 RNF6 NRP2 GAS7 CCDC64	IGF1R BDNF	DISC1 BDNF	SNAP25 CREB1 BDNF	BDNF	DISC1 YWHAH FZD3 SNAP25 BDNF	--

Neuron projection morphogenesis	GO:0048812	33,5906	52	0,0462928	GJA1 PAFAH1B1 IGF1R ADM APC FEZF1 BMPR1B CELSR2 ERBB3 NGF OPHN1 PTPRZ1 TIAM1 YWHAH ULK1 KALRN DMD VCAN DVL1 EPHA4 EPHA7 KIF5C POU4F2 DCLK1 CNTN2 POU3F2 SMAD1 TBR1 PIP5K1C CYFIP1 ULK2 RUFY3 NFASC FZD3 BAIAP2 SEMA6A LRRC4C NDEL1 SLITRK6 WNT3A SNAP25 CREB1 NRXN1 NRXN3 HOXA1 BDNF UNC5B DCX ATL1 RNF6 NRP2 GAS7	BDNF IGF1R	BDNF	SNAP25 CREB1 BDNF	BDNF	FZD3 SNAP25 BDNF YWHAH	--

This table lists interesting Gene Ontology (GO) subcategories and over-represented target genes with *P*values <0.05 and FDR adjustment related to nervous system development. Target genes associated with AD and other neurological diseases are listed separately.

AD: Alzheimer disease; BP: bipolar disorder; DEP: major depression; PD, Parkinson's disease; SCHIZ: schizophrenia

Target gene prediction for the two unknown target genes brain-miR-112 and brain-miR-161 revealed 234 target genes for brain-miR-112, but only six target genes for brain-miR-161. Over-representation analysis was done for both brain-miRNAs separately. Here, we identified 126 GO categories with *P*value <0.05 for brain-miR-112, with significant enrichment of miRNA targets in GO categories associated with nervous system and neuron function (see Table 4). For brain-miR-161 no significant GO categories were found.

**Table 4:** Results of the over-representation analysis of the predicted target genes of brain-miR-112.

Subcategory	Subcategory alternative name	Expected	Observed	*P*value (FDR)	Target genes	AD	BD	DEP	PD	SCHIZ	Multiple sclerosis
Neurogenesis	GO:0022008	119.612	31	0.000284421	ONECUT2 ANK3 CACNB3 CDK6 CELSR3 FGFR2 MEF2A NFIB PICALM PLAG1 PLXNA1 PSD4 PTPRR RAB11A RPS6KA4 SIX4 COL4A4 DFNB31 DISC1 SRF STX3 ADCY1 CDKN1C CNP ENAH HOXC10 LIF LRP6 ROCK1 RPS6KA3 TFAP2A	--	DISC1	--	--	DISC1	--

Neuron differentiation	GO:0030182	103.937	27	0.000801251	ONECUT2 ANK3 CACNB3 CELSR3 FGFR2 MEF2A NFIB PICALM PLXNA1 PSD4 PTPRR RAB11A RPS6KA4 COL4A4 DFNB31 SRF STX3 ADCY1 CDKN1C CNP ENAH HOXC10 LIF LRP6 ROCK1 RPS6KA3 TFAP2A	--	--	--	--	--	--

Neuron development	GO:0048666	845.125	23	0.00159317	ONECUT2 ANK3 CACNB3 CELSR3 FGFR2 MEF2A NFIB PICALM PLXNA1 RAB11A RPS6KA4 COL4A4 DFNB31 SRF STX3 ADCY1 CDKN1C CNP ENAH LIF ROCK1 RPS6KA3 TFAP2A	--	--	--	--	--	--

Nervous system development	GO:0007399	184.019	37	0.00268227	ONECUT2 ANK3 CACNB3 CDK6 CELSR3 FGFR2 MEF2A NFIB PICALM PLAG1 PLXNA1 PSD4 PTPRR RAB11A RPS6KA4 SEMA5B SIX4 COL4A4 DFNB31 DISC1 FGF1 SRF STX3 SULF1 ADCY1 ARHGEF15 CDKN1C CNP ENAH HOXC10 LIF LPHN1 LRP6 MEN1 ROCK1 RPS6KA3 TFAP2A	FGF1	DISC1	--	--	DISC1	--

Neuron projection development	GO:0031175	736.077	19	0.00946322	ANK3 CACNB3 CELSR3 FGFR2 MEF2A NFIB PICALM PLXNA1 RAB11A RPS6KA4 COL4A4 SRF STX3 ADCY1 CNP ENAH LIF ROCK1 RPS6KA3	--	--	--	--	--	--

Neuron projection	GO:0043005	633.844	16	0.0262424	ALOX5 ANK3 MYLK2 NFIB SLC38A7 DFNB31 DISC1 FRMPD4 GRIA4 SLC6A1 STX3 AAK1 ALDOC ARHGEF15 BACE1 LPHN1	BACE1	DISC1	--	--	DISC1 GRIA4	--

Neurotransmitter:sodium symporter activity	GO:0005328	0.215825	3	0.0330004	SLC6A20 SLC6A1 SLC6A6	--	--	--	--	--	--

Neuron projection morphogenesis	GO:0048812	624.756	15	0.0391162	ANK3 CACNB3 CELSR3 FGFR2 MEF2A NFIB PICALM PLXNA1 RPS6KA4 COL4A4 ADCY1 CNP ENAH ROCK1 RPS6KA3	--	--	--	--	--	--

Neurotransmitter transporter activity	GO:0005326	0.272621	3	0.0412241	SLC6A20 SLC6A1 SLC6A6	--	--	--	--	--	--

Neuroblast division	GO:0055057	0.0795144	2	0.0412241	FGFR2 LRP6	--	--	--	--	--	--

Forebrain neuroblast division	GO:0021873	0.0795144	2	0.0412241	FGFR2 LRP6	--	--	--	--	--	--

Generation of neurons	GO:0048699	112.797	31	0.000188727	ONECUT2 ANK3 CACNB3 CDK6 CELSR3 FGFR2 MEF2A NFIB PICALM PLAG1 PLXNA1 PSD4 PTPRR RAB11A RPS6KA4 SIX4 COL4A4 DFNB31 DISC1 SRF STX3 ADCY1 CDKN1C CNP ENAH HOXC10 LIF LRP6 ROCK1 RPS6KA3 TFAP2A	--	DISC1	--	--	DISC1	--

Cell morphogenesis involved in neuron differentiation	GO:0048667	616.805	15	0.0366659	ANK3 CACNB3 CELSR3 FGFR2 MEF2A NFIB PICALM PLXNA1 RPS6KA4 COL4A4 ADCY1 CNP ENAH ROCK1 RPS6KA3	--	--	--	--	--	--

This table lists interesting Gene Ontology (GO) subcategories with *P*values <0.05 and FDR adjustment related to nervous system development.

AD: Alzheimer disease; BP: bipolar disorder; DEP: major depression; PD, Parkinson's disease; SCHIZ: schizophrenia

## Discussion

At present, there is no single molecular test that is suitable to reliably diagnose AD with adequate specificity and sensitivity. Tests for the analysis of CSF proteins like Aß42 or tau have high specificity and sensitivity, but are only indicated as confirmation of AD diagnosis based on clinical symptoms or as differential diagnosis to differentiate between AD and other forms of diseases that can cause symptoms like dementia. The analysis of SNPs in certain genes (for example, ApoE) yields too low diagnostic accuracy and is therefore not recommended as diagnostic test for AD. Furthermore, Ray et al. yielded promising results by the identification of 18 proteins in blood plasma that could differentiate AD patients from controls with 90% accuracy [25].

Here, we investigate whether blood-borne miRNA expression signatures might contribute to AD diagnosis. Until now, many efforts have been made to understand the role of miRNAs in neurodegenerative disorders, as summarized by Eacker et al. [26]. However, there are only two publications dealing with the miRNA expression in peripheral blood mononuclear cells (PBMC) of AD patients. The study by Villa et al. analyzed the expression of heterogeneous nuclear ribonucleoprotein (hnRNP)-A1, that is involved in the maturation of APP mRNA, and showed that the decreased expression of hsa-miR-590-3p is negatively correlated with the increased hnRNP-A1 mRNA levels [27]. The study by Schipper et al. [28] investigated the expression of 462 different miRNAs in PBMCs of 16 AD patients and 16 healthy controls to identify miRNAs that are responsible for the regulation of transcription of mRNA species that were previously reported to be downregulated in PBMCs of AD patients [29]. Only a modest relative increase of miRNA expression in AD PBMC in the range of 1.1- to 1.4-fold was found for nine miRNAs, namely hsa-miR-34a, hsa-miR-579, hsa-miR-181b, hsa-miR-520h, hsa-miR-155, hsa-miR-517*, hsa-let-7f, hsa-miR-200a, and hsa-miR-371. These data link the development of AD pathology to systemic dysfunction in the cellular stress/antioxidant response and genomic maintenance [28].

Using high throughput sequencing, we identified 140 unique miRNAs from 180 precursors that were differentially expressed between whole blood obtained from AD patients and healthy controls. It is incumbent upon the investigator, who proposes a set of miRNAs as done here to examine whether there is any known connection of these miRNAs and their target genes to neurodegeneration. Below we discuss this aspect in respect to our findings of dys-regulated miRNAs in blood of AD patients compared to healthy controls.

According to our TAM analysis out of the downregulated miRNAs, six were associated with the disease category Alzheimer disease including hsa-miR-21, hsa-miR-17, hsa-miR-29a, hsa-miR-29b, hsa-miR-106b, and hsa-miR-107. In a mouse model, Wang et al. investigated the involvement of hsa-miR-106b in the TGF-β signaling pathway that plays a key role in the pathogenesis of AD and found an inverse correlation between the expression of hsa-miR-106b and TGF-β type II receptor (TβR II) protein level [30]. In addition, Hebert et al. showed that hsa-miR-106b affects the expression of Amyloid precursor protein (APP) *in vitro*. Furthermore, they found a statistically significant decrease in hsa-miR-106b expression in sporadic AD patients, but the correlation between miR-106b and APP expression in AD brain was not significant [31]. The same group showed an inverse correlation between increased BACE1 levels and decreased miR-29a/b-1 expression [15]. Shioya et al. also observed a decreased expression of hsa-miR-29a in brain tissue of AD patients [32]. They also identified neuron navigator 3 (NAV3), a regulator of axon guidance, as a target of hsa-miR-29a and found elevated NAV3 mRNA levels in AD brains [32]. Hsa-miR-17 was shown to regulate APP expression *in vitro *and under physiological conditions in cells [31,33]. MiR-21 was shown to be downregulated in time-course assays of mature murine primary hippocampal cell cultures after neuronal Aβ treatments [34].

We further performed over-representation analysis with the 2,354 predicted targets of the 10 known miRNAs of our 12-miRNA signature. Here, several GO categories, with significant enrichment of miRNA targets in the GO categories linked to the nervous system, were found. Most interestingly, some of these target genes have already been related to AD or other of the investigated neurological diseases. One of the most prominent examples is DRD1 that encodes the Dopamine receptor D1, which is the most abundant dopamine receptor in the central nervous system. DRD1 is associated with AD, BD, and SCHIZ. Another example, DISC1 (disrupted in SCHIZ), associated with BD and SCHIZ, encodes a protein involved in neurite outgrowth and cortical development. BDNF (brain-derived neurotrophic factor) important for survival of striatal neurons in the brain is known to be downregulated in AD patients and also associated with BD, DEP, PD, and SCHIZ. IGF1R is the only target gene that was exclusively found to be associated with AD. The protein encoded by this gene is increased in temporal cortex surrounding and within Aß-containing plaques, but a significantly lower number of neurons of AD patients express IGF1R [35]. This suggests that IGF1R signaling normally controlling vital growth, survival, and metabolic functions in the brain is disturbed in AD brains. The two unknown miRNAs revealed 234 target genes for brain-miR-112, but only six target genes for brain-miR-161. In the over-representation analysis for brain-miR-112 we also identified GO categories linked to the nervous system, including targets like DISC1 as discussed above. For brain-miR-161 we found no significant GO categories. However, a literature review of the six target genes of brain-miR-161 revealed some interesting findings. GRID1 (glutamate receptor, ionotropic, delta 1), predicted to be a target gene of brain-miR-161, encodes a gene product that is a subunit of glutamate receptor channels which mediate most of the fast excitatory synaptic transmission in the central nervous system and play key roles in synaptic plasticity. Interestingly, GRID1 has previously been associated with SCHIZ and BD [36-38]. Another predicted target gene CCDN2 (Cyclin D2) plays a role in corticogenesis [39].

However, we have to point out that our analysis is based on whole blood. Previous findings on cancer suggest that the miRNA expression pattern between blood cells and cancer tissue do not necessarily show the same expression pattern but some overlaps can be found [40-42]. Unfortunately, tissue and blood samples of the same patients were not available for the present study. Nevertheless, we performed database analysis and extracted all miRNAs deregulated in AD and the corresponding literature out of the Human MiRNA& Disease Database [43]. In total, we found 18 different publications, with 15 publications on AD brain tissue and/or cell culture models. Out of those studies, 29 different miRNAs deregulated in AD are listed in the HMDD. Comparing these miRNAs with our data revealed eight of the 29 miRNAs that were significantly dys-regulated in blood cells in our study. There is, however, no evidence whether these overlaps were found by chance or not. Any link between deregulated miRNAs in blood of patients with neurological diseases and the disease itself has to be considered with caution.

Since a large set of miRNAs often contains a significant amount of redundant miRNAs with largely identical information content the differentiation between AD samples and healthy controls using a reduced set of miRNAs may likely yield comparably accurate results. Therefore, a panel of 12 miRNAs with limited cross-correlation, including most strongly dys-regulated miRNAs that show a potential to separate AD from controls, was selected. Some of these 12 miRNAs have already been related to AD. For example, Wang et al. showed in a computational analysis that the 3'-untranslated region (UTR) of beta-site amyloid precursor protein-cleaving enzyme 1 (BACE1) mRNA is targeted by hsa-miR-107 and that BACE1 mRNA levels tended to increase as miR-107 levels decreased in the progression for AD. An increased BACE1 expression is an important risk factor for sporadic AD [15]. Nelson et al. also showed a negative correlation between the expression of hsa-miR-107 and BACE1 [44]. Interestingly, hsa-miR-107 that was also part of our 12-miRNA signature investigated in the presented study was also downregulated in blood of AD patients compared to healthy controls. Augustin et al. [45] recently investigated miRNAs that are predicted to target another AD-related gene, namely ADAM10, which controls the proteolytic processing of *APP *and the formation of the amyloid plaques. Database analyses prompted them to further investigate two miRNAs that were also included in our 12-miRNA signature, namely hsa-miR-107 and hsa-miR-103. They found that predicted target genes of hsa-miR-107 and hsa-miR-103 showed significant overlap with the AlzGene database. In a reporter assay ADAM10 expression was reduced by both miRNAs. These two miRNAs were also investigated in relation to the expression of cofilin protein in a transgenic mouse model [46]. Cofilin binds to actin resulting in the formation of Hirano bodies, which may play an essential role in AD pathogenesis. In APP transgenic mouse brains hsa-miR-107 and hsa-miR-103 levels were decreased while cofilin levels were increased and in a luciferase assay it was demonstrated that hsa-miR-107 and hsa-miR-103 were able to reduce the expression of cofilin. In our RT-qPCR approach both miRNAs hsa-miR-107 and hsa-miR-103 showed the same expression pattern, that is, both were downregulated in blood of AD, PD, DEP, and SCHIZ patients and upregulated in mild cognitive impairment, multiple sclerosis, and BD patients. All other miRNAs of our 12-miRNA signature have not been identified or investigated so far in relationship to AD.

While we showed the 12-miRNA signature's potential to separate AD patients from controls with an accuracy of 93.3%, we also tested its applicability as differential diagnostic biomarker to separate AD from other neurological diseases. As we expected, the accuracy decreased when trying to use this signature for separating other neurodegenerative diseases from controls or separating AD from other neurological disorders. Remarkably, the classification of psychiatric disorders *versus *controls yielded an even better accuracy than for AD *versus *controls. These findings suggest a relevance of the considered 12 miRNAs also for psychological disorders. The association of the 12-miRNA signature with neurological diseases in general is further underlined by the results of our over-representation analysis using GeneTrail. Here, we found four significant GO categories related to nervous system and neurons with an over-representation of target genes of the 10 known miRNAs from our 12-miRNA signature. In addition, out of the 10 known miRNAs nine miRNAs are already included in the HMDD and five of those miRNAs that were previously associated with neurological diseases including AD, PD, and SCHIZ. As mentioned above, Yao et al. [46] showed that reduced levels of hsa-miR-103 or hsa-miR-107 are associated with elevated cofilin protein levels and formation of rod-like structures in a transgenic mouse model of AD. Both miRNAs were also downregulated in our study. Martins et al. [47] showed that hsa-miR-151a-3p and hsa-miR-26a-5p are differentially expressed in PBMCs (peripheral blood mononuclear cells) of PD patients and controls. In prefrontal cortex tissue of individuals with SCHIZ hsa-miR-26b was downregulated [48]. Target analysis of the miRNA that was not included in HMDD, hsa-miR-5010-3p, revealed target genes involved in nervous system processes. For example, predicted targets of hsa-miR-5010-3p include the NFASC (neurofascin), that functions in neurite outgrowth, and organization of nodes of Ranvier on axons, NPY (Neuropeptide Y), that is one of the most abundant neuropeptides in the mammalian central nervous system [49], NLGN1 (neuroligin 1), that may be involved in the formation and remodeling of central nervous system synapses, NRXN3 (neurexin3), that functions in the nervous system as receptors and cell adhesion molecule, and NCAN (neurocan), that seems to be a genetic risk factor for BD.

Finally, one has to take into account that AD is a complex progressive neurodegenerative disease causing cognitive, behavioral, and functional problems that are also found in other neurological diseases. Furthermore, dementia is not only caused by AD but can result from other neurological disorders. Dementia patients often suffer from other additional mental and behavioral problems like depression, anxiety, psychosis, agitation, and aggression further complicating correct classification. As AD shares common neuropsychiatric symptoms with other neurological diseases there might be an overlap with the associated medication.

Most importantly one needs to point out that as the patients included in our study are not treatment-naïve, we cannot exclude the influence of administered drugs on the miRNA signature. As an example, Bocchio-Chiavetto et al. showed that chronic anti-depressant treatment has effects on the blood miRNA profile [50]. Furthermore, we have to point out that we do not have a birth cohort. Nevertheless, the age distribution between the AD samples and the control samples used for NGS is not significantly different (*P*value 0.1147). The age distribution of AD patients, MCI patients, PD patients, and controls is quite similar. Patients suffering from multiple sclerosis, DEP, BD, or SCHIZ are about 20 to 30 years younger. The differences in the age distribution are due to the differences between the onsets of the diseases. In previous studies [51] we already investigated the influence of age and gender on the miRNA expression profile of whole blood. We did not find any statistically significant deregulated miRNAs between men and women. The miRNA with the lowest *P*value was hsa-miR-423 (*P*value 0.78). To test for the influence of age we compared the profiles obtained from old *versus *young patients by splitting the total group in half based on the age. Here, the miRNA with the lowest *P*value was hsa-miR-890 (*P*value 0.87). Again, we did not find any deregulated miRNAs. In summary, we found no evidence that age and gender have a substantial influence on the miRNA profiles. Both miRNAs mentioned above were not significant in the present study on AD.

## Conclusion

Here we identified 140 unique differentially expressed miRNAs between AD patients and healthy controls. Using a signature of 12 miRNAs differentially expressed between AD patients and healthy controls we were not only able to distinguish with high diagnostic accuracies between AD patients and healthy controls, but also between AD patients and patients suffering from other neurological disorders including mild cognitive impairment as a potential preliminary stage of AD, and other neurodegenerative diseases like PD and multiple sclerosis as well as mental diseases like SCHIZ, DEP, and BD. However, additional work will be needed to elucidate the applicability of this 12-miRNA signature as a potential diagnostic test for AD and the above-mentioned effects of the drug treatments commonly used in the treatment of the disease. Hopefully, tests of this non-invasive and relatively cheap kind will be applicable to prodromal AD cases and to MCI patients with the aim to recognize early AD to initiate treatment.

## Materials and methods

### Patient details

We analyzed the expression of miRNAs in peripheral blood of a total of 215 patients and healthy controls, either by NGS or by RT-qPCR or by both methods (see Table 1). In detail, we obtained 2.5 mL blood collected in PAXgene Blood RNA tubes (PreAnalytiX) from patients with AD (*n *= 106), patients with mild cognitive impairment (MCI) (*n *= 18), patients with multiple sclerosis (clinically isolated syndrome, CIS) (*n *= 16), patients with PD (*n *= 9), patients with DEP (*n *= 15), patients with BD (*n *= 15), patients with SCHIZ (*n *= 14), and from healthy controls (C) (*n *= 22). Samples from patients with AD stem from the Biorepository and Tissue Bank PrecisionMed (San Diego, CA, USA) (*n *= 97) and the University Clinic of Erlangen (Germany) (*n *= 9), samples from healthy controls and from patients with MCI, PD, DEP, BD, and SCHIZ stem from PrecisionMed (San Diego, CA, USA) and samples from patients with CIS stem from Charité Berlin (Germany). Detailed patient characteristics are listed in Additional file 4-Table S3. AD and MCI patients were diagnosed by using state of the art criteria. In detail, in order to be included in the 'probable AD' group, patients fulfilled the following criteria of the NINCDS-ADRDA (National Institute of Neurological and Communicative Disorders and Stroke and the Alzheimer disease and Related Disorders Association) [52]: MMSE >14 and <26, deficit in two or more areas of cognition, progressive worsening of memory and other cognitive functions, no disturbance of consciousness, onset between the ages of 40 and 90 years, most often after 65 years, and absence of systemic disorders or other brain diseases that could account for the progressive deterioration in cognition. Furthermore, MRI or CT reports that were compatible with AD are available. The median MMSE score for the AD patients was 18.9 (3.4).

Samples included in the MCI group fulfilled the following criteria: MMSE >22 and <28, not demented, memory complaint, preserved general cognitive function, intact activities of daily living: (allowed problems with 2 or less of the following: phone calls, meal preparation, handling money, completing chores), abnormal memory function documented by scoring below the education adjusted cutoff on the Logical Memory II subscale (delayed paragraph recall) from the Wechsler Memory Scale-Revised (maximum score = 25) with (a) <8 for 16 years or more of education, (b) <4 for 8-15 years of education, (c) <2 for 0-7 years of education. The median MMSE score for the MCI patients was 25.3 (±1.4).

The study was approved by the institutional review boards of Charité - Universitätsmedizin Berlin (EA1/182/10) and the study was performed in accordance with the Helsinki declaration. Written informed consent was obtained from all patients participating in the study.

Samples and clinical data supplied by PrecisionMed are handled in strictest compliance with all applicable rules and regulations including the recommendations of the Council of the Human Genome Organization (HUGO) Ethical, Legal, and Social Issues Committee (HUGO-ELSI, 1998); with the United Nations Educational, Scientific, and Cultural Organization's (UNESCO) Universal Declaration on the Human Genome and Human Rights (1997); and with recommendations guiding physicians in biomedical research involving human subjects adopted by the 18th World Medical Assembly, Helsinki, Finland, 1964 and later revisions.

### RNA isolation

Total RNA including miRNA was isolated using the PAXgene Blood miRNA Kit (Qiagen) following the manufacturer's recommendations. Isolated RNA was stored at -80°C until use. RNA integrity was analyzed using Bioanalyzer 2100 (Agilent) and concentration and purity were measured using NanoDrop 2000 (Thermo Scientific).

### Library preparation and next-generation sequencing

We first analyzed samples from AD patients (*n *= 48) and healthy controls (*n *= 22) by NGS.

For the library preparation, 200 ng of total RNA was used per sample, as determined with a RNA 6000 Nano Chip on the Bioanalyzer 2100 (Agilent). Preparation was performed following the protocol of the TruSeq Small RNA Sample Prep Kit (Illumina). Concentration of the ready prepped libraries was measured on the Bioanalyzer using the DNA 1000 Chip. Libraries were then pooled in batches of six samples in equal amounts and clustered with a concentration of 9 pmol in one lane each of a single read flowcell using the cBot (Illumina). Sequencing of 50 cycles was performed on a HiSeq 2000 (Illumina). Demultiplexing of the raw sequencing data and generation of the fastq files was done using CASAVA v.1.8.2.

### NGS data analysis

The raw Illumina reads were first preprocessed by cutting the 3' adapter sequence using the program fastx_clipper from the FASTX-Toolkit [53]. Reads shorter than 18 nts after clipping were removed. The remaining reads are reduced to unique reads and their frequency per sample to make the mapping steps more time efficient. For the remaining steps, we used the miRDeep2 pipeline [54]. These steps consist of mapping the reads against the genome (hg19), mapping the reads against miRNA precursor sequences from miRBase release v18, summarizing the counts for the samples, and the prediction of novel miRNAs. Since the miRDeep2 pipeline predicts in our case the novel miRNAs per sample, we merged the miRNAs afterwards as follows: first, we extract the novel miRNAs per sample that have a signal-to-noise ratio >10. Subsequently, we merge only those novel miRNAs that are located on the same chromosome, and both their mature forms share an overlap of at least 11 nucleotides. The remaining putative novel miRNAs were mapped with BLAST (v 2.2.24, [55]) against known ncRNA and miRNA sequences from diverse sources (miRBase v18 [56], snoRNA-LBME-db [57], ncRNAs from Ensembl 'Homo_sapiens.GRCh37.67.ncrna.fa'[58], NONCODE v3.0[59]). We excluded sequences that aligned with >90% of their length (allowing 1 mismatch) to any of the ncRNA sequences. All NGS data are publicly available in GEO database (GSE46579 [60]).

### Bioinformatics analysis

For the NGS analysis, we excluded miRNAs with <50 read counts summed up across all samples of each group (AD or control), since these were considered lowly abundant. We normalized the read counts using standard quantile normalization. Next, we calculated for each miRNA the area under the receiver operator characteristic curve (AUC), the fold-change, and the significance value (*P*value) using Wilcoxon-Mann-Whitney (WMW) test. All significance values were adjusted for multiple testing using the Benjamini-Hochberg approach [61,62]. The bioinformatics analyses have been carried out using the freely available tool R [63]. For classification purposes, we used support vector machines (SVM) from the R package e1071. If not stated otherwise, we computed the group-wise classifications using linear kernels in 10-fold cross-validations with 100 repetitions. In addition, we computed the classification of permuted class labels with the same parameters as control. If group sizes were unbalanced, we randomly selected samples from the bigger group to match the sample sizes in the smaller group in each repetition.

### Database analysis

MiRNA enrichment analysis was performed using the TAM tool [23,24]. The miRNA targets of the known miRNAs were predicted using miRDB [64-66]. Targets for the unknown brain-miRs were predicted using TargetScan [67,68]. TargetScan is able to predict targets of miRBase miRNAs as well as targets of other sequences by using the heptamer seed sequence (nucleotides 2-8) of a potential miRNA. For brain-miR-161 we used the heptamer UUCGAAA, for brain-mir-112 GCUCUGU. With the predicted miRNA target genes we performed an over-representation analysis using the gene set analysis tool GeneTrail [69,70] with default settings. The *P*values for the biological categories were adjusted by False Discovery Rate (FDR) [71] and were considered significant if <0.05. Furthermore, we searched for miRNA-disease interactions using the Human MiRNA& Disease Database (HMDD [43,72]).

### Quantitative real time-PCR (RT-qPCR)

For validation purposes we analyzed the expression of single miRNAs using quantitative real time-polymerase chain reaction (RT-qPCR) in the same samples as used for NGS, if sufficient amounts of RNA were available. We used the miScript PCR System (Qiagen) for reverse transcription and RT-qPCR. A total of 200 ng RNA was converted into cDNA using the miScript Reverse Transcription Kit according to the manufacturer's protocol. The RT-qPCR was performed with the miScript SYBR® Green PCR Kit in a total volume of 20 μL per reaction containing 1 μL cDNA according to the manufacturer's protocol. For each miScript Primer Assay we additionally prepared a PCR negative-control with water instead of cDNA (non-template control).

We further expanded the number of samples by further samples from patients with AD, MCI, CIS, PD, DEP, BD, and SCHIZ, resulting in a total of 202 samples analyzed by RT-qPCR (see Table 1). In detail, we analyzed with RT-qPCR a total of 94 samples from AD patients, 18 samples from MCI patients, 16 samples from CIS patients, nine samples from PD patients, 15 samples from DEP patients, 15 samples from BD patients, 14 samples from SCHIZ patients, and 21 samples from healthy controls.

Out of the NGS results we selected 12 miRNAs deregulated between patients with AD and healthy individuals. The set contained the following miRNAs: The upregulated miRNAs brain-miR-112, brain-miR-161, hsa-let-7d-3p, hsa-miR-5010-3p, hsa-miR-26a-5p, hsa-miR-1285-5p, and hsa-miR-151a-3p as well as the downregulated miRNAs hsa-miR-103a-3p, hsa-miR-107, hsa-miR-532-5p, hsa-miR-26b-5p, and hsa-let-7f-5p, respectively.

While 10 of the 12 miRNAs have already been annotated in the miRBase, two miRNAs, namely brain-miR-112 and brain-miR-161, were newly identified and not yet included in miRBase [21,22]. As endogenous control we used the small nuclear RNA RNU48.

## List of abbreviations

ACE: Aging Cognition Evaluation; AD: Alzheimer disease; ADAS-Cog: Alzheimer disease Assessment Scale-cognitive subscale; AUC: area under the receiver operator characteristics curve; BD: bipolar disorder; C: healthy control; CDR: Clinical Dementia Rating; CIS: clinically isolated syndrome; CLIA: Clinical Laboratory Improvement Amendments; CNS: central nervous system; CSF: cerebrospinal fluid; DEP: major depression; FDR: False Discovery Rate; GO: Gene Ontology; HMDD: Human MiRNA& Disease Database; HUGO: Human Genome Organization; MCI: mild cognitive impairment; miRNA: micro Ribo Nucleic Acid; MMSE: Mini-Mental State Exam; mtDNA: mitochondrial DNA; NGS: next generation sequencing; PBMC: peripheral blood mononuclear cells; PD: Parkinson disease; RT-qPCR: quantitative Real Time Polymerase Chain Reaction; SAMPLE: Serial Alzheimer disease and MCI Prospective Longitudinal Evaluation; SCHIZ: schizophrenia; SNP: single nucleotide polymorphisms; SVM: support vector machines; TAM: tool for annotations of human miRNAs; TF: transcription factor; UBC: University of British Columbia; UCSC: University of California Santa Cruz; UNESCO: United Nations Educational, Scientific, and Cultural Organization; WMW: Wilcoxon-Mann-Whitney.

## Competing interests

AK and CS are employees of Siemens Healthcare. Siemens Healthcare in part supported this work.

## Authors' contributions

The work presented here was carried out in collaboration between all authors. EM, AK, and CS initiated the study. EM, AK, and BM designed the study and KR and FP participated in the design of the study. PL, SD, KS, JH, KF, and BM performed the laboratory experiments. CB and AK analyzed and interpreted the data. SM assisted in data analysis. CL provided the clinical samples collected in Erlangen. PL and CB performed database analysis. TB interpreted the data and revised the manuscript critically. PL, CB, AK and EM wrote the paper. All authors read and approved the final manuscript.

## Supplementary Material

Additional file 1 **Table S1**. Table listing the 180 significantly dys-regulated miRNAs (140 unique mature miRNAs). Click here for file

Additional file 2 **Figure S1**. Heatmap for the 180 miRNAs significantly dys-regulated in AD patients compared to control individuals. Click here for file

Additional file 3 **Table S2**. Table listing all novel mature miRNAs. Click here for file

Additional file 4 **Table S3**. Table listing patient characteristics and indicates which samples are included in NGS analysis and/or in the RT-qPCR. Click here for file
