# Supplementary material for: A blood based 12-miRNA signature of Alzheimer disease patients
Source: Genome Biol. 2013 Jul 29;14(7):R78. doi: 10.1186/gb-2013-14-7-r78 (PMC4053778; doi:10.1186/gb-2013-14-7-r78)
Supplement: Additional file 2 — Figure S1. Heatmap for the 180 miRNAs significantly dys-regulated in AD patients compared to control individuals. [file gb-2013-14-7-r78-S2.PDF]

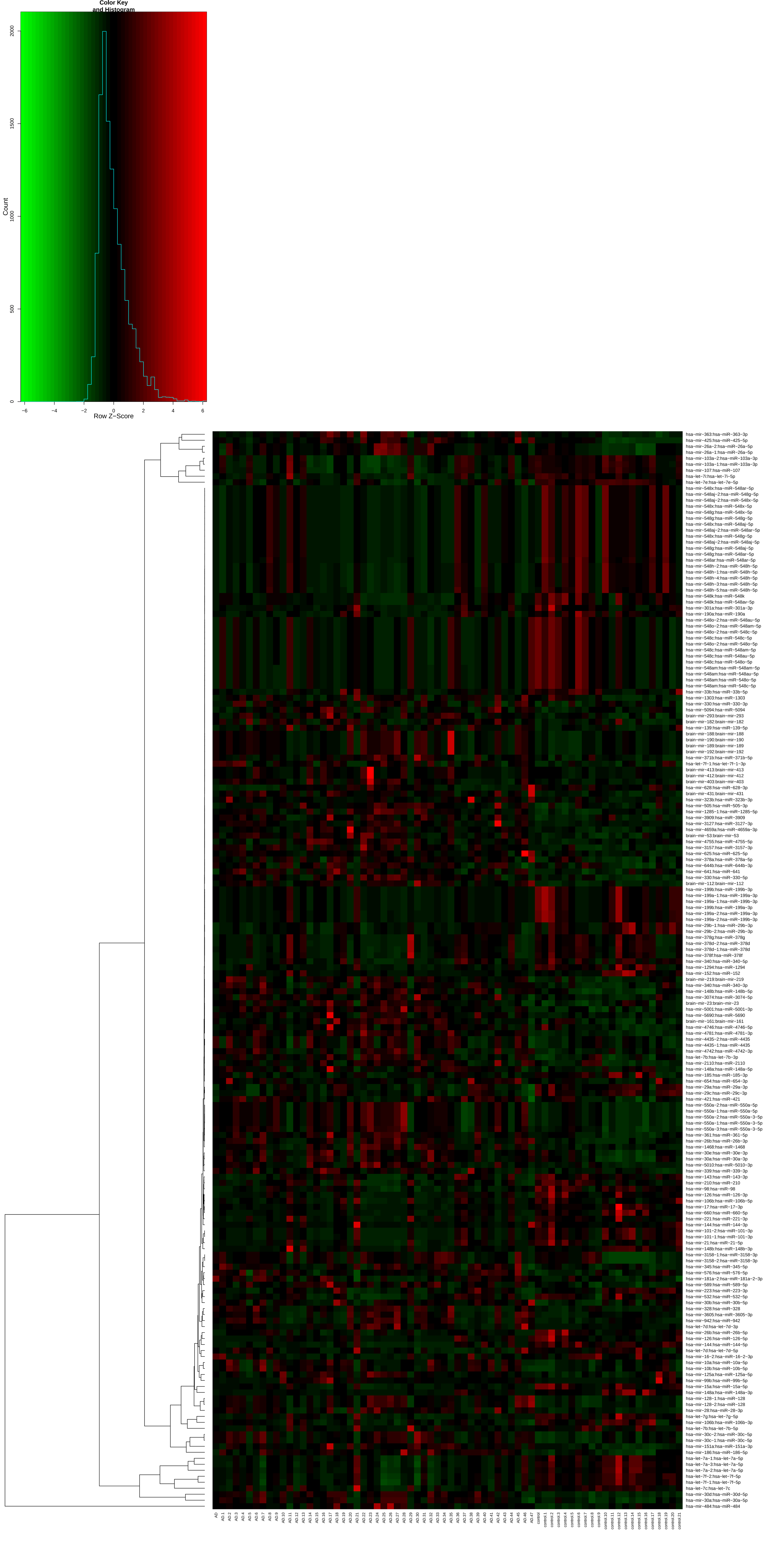
Color Key
and Histogram
Count
0
500
1000
1500
2000
−6
−4
−2
0
2
4
6
Row Z−Score
hsa−mir−363:hsa−miR−363−3p
hsa−mir−425:hsa−miR−425−5p
hsa−mir−26a−2:hsa−miR−26a−5p
hsa−mir−26a−1:hsa−miR−26a−5p
hsa−mir−103a−2:hsa−miR−103a−3p
hsa−mir−103a−1:hsa−miR−103a−3p
hsa−mir−107:hsa−miR−107
hsa−let−7i:hsa−let−7i−5p
hsa−let−7e:hsa−let−7e−5p
hsa−mir−548x:hsa−miR−548ar−5p
hsa−mir−548aj−2:hsa−miR−548g−5p
hsa−mir−548aj−2:hsa−miR−548x−5p
hsa−mir−548x:hsa−miR−548x−5p
hsa−mir−548g:hsa−miR−548x−5p
hsa−mir−548g:hsa−miR−548g−5p
hsa−mir−548x:hsa−miR−548aj−5p
hsa−mir−548aj−2:hsa−miR−548ar−5p
hsa−mir−548x:hsa−miR−548g−5p
hsa−mir−548aj−2:hsa−miR−548aj−5p
hsa−mir−548g:hsa−miR−548aj−5p
hsa−mir−548g:hsa−miR−548ar−5p
hsa−mir−548ar:hsa−miR−548ar−5p
hsa−mir−548h−2:hsa−miR−548h−5p
hsa−mir−548h−1:hsa−miR−548h−5p
hsa−mir−548h−4:hsa−miR−548h−5p
hsa−mir−548h−3:hsa−miR−548h−5p
hsa−mir−548h−5:hsa−miR−548h−5p
hsa−mir−548k:hsa−miR−548k
hsa−mir−548k:hsa−miR−548av−5p
hsa−mir−301a:hsa−miR−301a−3p
hsa−mir−190a:hsa−miR−190a
hsa−mir−548o−2:hsa−miR−548au−5p
hsa−mir−548o−2:hsa−miR−548am−5p
hsa−mir−548o−2:hsa−miR−548c−5p
hsa−mir−548c:hsa−miR−548c−5p
hsa−mir−548o−2:hsa−miR−548o−5p
hsa−mir−548c:hsa−miR−548am−5p
hsa−mir−548c:hsa−miR−548au−5p
hsa−mir−548c:hsa−miR−548o−5p
hsa−mir−548am:hsa−miR−548am−5p
hsa−mir−548am:hsa−miR−548au−5p
hsa−mir−548am:hsa−miR−548o−5p
hsa−mir−548am:hsa−miR−548c−5p
hsa−mir−33b:hsa−miR−33b−5p
hsa−mir−1303:hsa−miR−1303
hsa−mir−330:hsa−miR−330−3p
hsa−mir−5094:hsa−miR−5094
brain−mir−293:brain−mir−293
brain−mir−182:brain−mir−182
hsa−mir−139:hsa−miR−139−5p
brain−mir−188:brain−mir−188
brain−mir−190:brain−mir−190
brain−mir−189:brain−mir−189
brain−mir−192:brain−mir−192
hsa−mir−371b:hsa−miR−371b−5p
hsa−let−7f−1:hsa−let−7f−1−3p
brain−mir−413:brain−mir−413
brain−mir−412:brain−mir−412
brain−mir−403:brain−mir−403
hsa−mir−628:hsa−miR−628−3p
brain−mir−431:brain−mir−431
hsa−mir−323b:hsa−miR−323b−3p
hsa−mir−505:hsa−miR−505−3p
hsa−mir−1285−1:hsa−miR−1285−5p
hsa−mir−3909:hsa−miR−3909
hsa−mir−3127:hsa−miR−3127−3p
hsa−mir−4659a:hsa−miR−4659a−3p
brain−mir−53:brain−mir−53
hsa−mir−4755:hsa−miR−4755−5p
hsa−mir−3157:hsa−miR−3157−3p
hsa−mir−625:hsa−miR−625−5p
hsa−mir−378a:hsa−miR−378a−5p
hsa−mir−644b:hsa−miR−644b−3p
hsa−mir−641:hsa−miR−641
hsa−mir−330:hsa−miR−330−5p
brain−mir−112:brain−mir−112
hsa−mir−199b:hsa−miR−199b−3p
hsa−mir−199a−1:hsa−miR−199a−3p
hsa−mir−199a−1:hsa−miR−199b−3p
hsa−mir−199b:hsa−miR−199a−3p
hsa−mir−199a−2:hsa−miR−199a−3p
hsa−mir−199a−2:hsa−miR−199b−3p
hsa−mir−29b−1:hsa−miR−29b−3p
hsa−mir−29b−2:hsa−miR−29b−3p
hsa−mir−378g:hsa−miR−378g
hsa−mir−378d−2:hsa−miR−378d
hsa−mir−378d−1:hsa−miR−378d
hsa−mir−378f:hsa−miR−378f
hsa−mir−340:hsa−miR−340−5p
hsa−mir−1294:hsa−miR−1294
hsa−mir−152:hsa−miR−152
brain−mir−219:brain−mir−219
hsa−mir−340:hsa−miR−340−3p
hsa−mir−148b:hsa−miR−148b−5p
hsa−mir−3074:hsa−miR−3074−5p
brain−mir−23:brain−mir−23
hsa−mir−5001:hsa−miR−5001−3p
hsa−mir−5690:hsa−miR−5690
brain−mir−161:brain−mir−161
hsa−mir−4746:hsa−miR−4746−5p
hsa−mir−4781:hsa−miR−4781−3p
hsa−mir−4435−2:hsa−miR−4435
hsa−mir−4435−1:hsa−miR−4435
hsa−mir−4742:hsa−miR−4742−3p
hsa−let−7b:hsa−let−7b−3p
hsa−mir−2110:hsa−miR−2110
hsa−mir−148a:hsa−miR−148a−5p
hsa−mir−185:hsa−miR−185−3p
hsa−mir−654:hsa−miR−654−3p
hsa−mir−29a:hsa−miR−29a−3p
hsa−mir−29c:hsa−miR−29c−3p
hsa−mir−421:hsa−miR−421
hsa−mir−550a−2:hsa−miR−550a−5p
hsa−mir−550a−1:hsa−miR−550a−5p
hsa−mir−550a−2:hsa−miR−550a−3−5p
hsa−mir−550a−1:hsa−miR−550a−3−5p
hsa−mir−550a−3:hsa−miR−550a−3−5p
hsa−mir−361:hsa−miR−361−5p
hsa−mir−26b:hsa−miR−26b−3p
hsa−mir−1468:hsa−miR−1468
hsa−mir−30e:hsa−miR−30e−3p
hsa−mir−30a:hsa−miR−30a−3p
hsa−mir−5010:hsa−miR−5010−3p
hsa−mir−339:hsa−miR−339−3p
hsa−mir−143:hsa−miR−143−3p
hsa−mir−210:hsa−miR−210
hsa−mir−98:hsa−miR−98
hsa−mir−126:hsa−miR−126−3p
hsa−mir−106b:hsa−miR−106b−5p
hsa−mir−17:hsa−miR−17−3p
hsa−mir−660:hsa−miR−660−5p
hsa−mir−221:hsa−miR−221−3p
hsa−mir−144:hsa−miR−144−3p
hsa−mir−101−2:hsa−miR−101−3p
hsa−mir−101−1:hsa−miR−101−3p
hsa−mir−21:hsa−miR−21−5p
hsa−mir−148b:hsa−miR−148b−3p
hsa−mir−3158−1:hsa−miR−3158−3p
hsa−mir−3158−2:hsa−miR−3158−3p
hsa−mir−345:hsa−miR−345−5p
hsa−mir−576:hsa−miR−576−5p
hsa−mir−181a−2:hsa−miR−181a−2−3p
hsa−mir−589:hsa−miR−589−5p
hsa−mir−223:hsa−miR−223−3p
hsa−mir−532:hsa−miR−532−5p
hsa−mir−30b:hsa−miR−30b−5p
hsa−mir−328:hsa−miR−328
hsa−mir−3605:hsa−miR−3605−3p
hsa−mir−942:hsa−miR−942
hsa−let−7d:hsa−let−7d−3p
hsa−mir−26b:hsa−miR−26b−5p
hsa−mir−126:hsa−miR−126−5p
hsa−mir−144:hsa−miR−144−5p
hsa−let−7d:hsa−let−7d−5p
hsa−mir−16−2:hsa−miR−16−2−3p
hsa−mir−10a:hsa−miR−10a−5p
hsa−mir−10b:hsa−miR−10b−5p
hsa−mir−125a:hsa−miR−125a−5p
hsa−mir−99b:hsa−miR−99b−5p
hsa−mir−15a:hsa−miR−15a−5p
hsa−mir−148a:hsa−miR−148a−3p
hsa−mir−128−1:hsa−miR−128
hsa−mir−128−2:hsa−miR−128
hsa−mir−28:hsa−miR−28−3p
hsa−let−7g:hsa−let−7g−5p
hsa−mir−106b:hsa−miR−106b−3p
hsa−let−7b:hsa−let−7b−5p
hsa−mir−30c−2:hsa−miR−30c−5p
hsa−mir−30c−1:hsa−miR−30c−5p
hsa−mir−151a:hsa−miR−151a−3p
hsa−mir−186:hsa−miR−186−5p
hsa−let−7a−1:hsa−let−7a−5p
hsa−let−7a−3:hsa−let−7a−5p
hsa−let−7a−2:hsa−let−7a−5p
hsa−let−7f−2:hsa−let−7f−5p
hsa−let−7f−1:hsa−let−7f−5p
hsa−let−7c:hsa−let−7c
hsa−mir−30d:hsa−miR−30d−5p
hsa−mir−30a:hsa−miR−30a−5p
hsa−mir−484:hsa−miR−484
AD
AD.1
AD.2
AD.3
AD.4
AD.5
AD.6
AD.7
AD.8
AD.9
AD.10
AD.11
AD.12
AD.13
AD.14
AD.15
AD.16
AD.17
AD.18
AD.19
AD.20
AD.21
AD.22
AD.23
AD.24
AD.25
AD.26
AD.27
AD.28
AD.29
AD.30
AD.31
AD.32
AD.33
AD.34
AD.35
AD.36
AD.37
AD.38
AD.39
AD.40
AD.41
AD.42
AD.43
AD.44
AD.45
AD.46
AD.47
control
control.1
control.2
control.3
control.4
control.5
control.6
control.7
control.8
control.9
control.10
control.11
control.12
control.13
control.14
control.15
control.16
control.17
control.18
control.19
control.20
control.21
